# Ruminative Tendency Relates to Ventral Striatum Functionality: Evidence From Task and Resting-State fMRI

**DOI:** 10.3389/fpsyt.2020.00067

**Published:** 2020-02-20

**Authors:** Alon Erdman, Rany Abend, Itamar Jalon, Moran Artzi, Tomer Gazit, Keren Avirame, Ezequiel Diego Ais, Hilik Levokovitz, Eva Gilboa-Schechtman, Talma Hendler, Eiran Vadim Harel

**Affiliations:** ^1^ Sagol Brain Institute Tel Aviv, Tel Aviv Sourasky Medical Center, Tel Aviv, Israel; ^2^ Psychology Department, Bar-Ilan University, Ramat Gan, Israel; ^3^ Section on Development and Affective Neuroscience, National Institute of Mental Health, Bethesda, MD, United States; ^4^ School of Psychological Science, Tel Aviv University, Tel Aviv, Israel; ^5^ Faculty of Medicine, Tel Aviv University, Tel Aviv, Israel; ^6^ Beer Yaakov Mental Health Center, affiliated with the Sackler Faculty of Medicine, Tel Aviv University, Tel Aviv, Israel; ^7^ Sagol School of Neuroscience, Tel Aviv University, Tel Aviv, Israel

**Keywords:** rumination, nucleus accumbens, ventral striatum, reward, depression

## Abstract

**Background:**

Ruminative responding involves repetitive and passive thinking about one’s negative affect. This tendency interferes with initiation of goal-directed rewarding strategies, which could alleviate depressive states. Such reward-directed response selection has been shown to be mediated by ventral striatum/nucleus accumbens (VS/NAcc) function. However, to date, no study has examined whether trait rumination relates to VS/NAcc functionality. Here, we tested whether rumination moderates VS/NAcc function both in response to reward and during a ruminative state.

**Methods:**

Trait rumination was considered dimensionally using Rumination Response Scale (RRS) scores. Our sample (N = 80) consisted of individuals from a community sample and from patients diagnosed with major depressive disorder, providing a broad range of RRS scores. Participants underwent fMRI to assess two modes of VS/NAcc functionality: 1) in response to reward, and 2) during resting-state, as a proxy for ruminative state. We then tested for associations between RRS scores and VS/NAcc functional profiles, statistically controlling for overall depressive symptom severity.

**Results:**

RRS scores correlated positively with VS/NAcc response to reward. Furthermore, we noted that higher RRS scores were associated with increased ruminative-dependent resting-state functional connectivity of the VS/NAcc with the left orbitofrontal cortex.

**Conclusions:**

These findings suggest that ruminative tendencies manifest in VS/NAcc reward- and rumination-related functions, providing support for a theoretical-clinical perspective of rumination as a habitual impairment in selection of rewarding, adaptive coping strategies.

## Introduction

Rumination is conceptualized as a mode of responding to distress, involving repetitive and passive thinking about one’s negative affect (1). Evidence indicates that the tendency to ruminate reliably predicts the development of sub-clinical and clinical depressive symptoms (2, 3). Although mainly studied in the context of major depressive disorder (MDD), rumination has been recognized as part of a transdiagnostic pattern of repetitive negative thinking, suggesting that it involves a range of cognitive processes such as repetitiveness, automatization, and intrusiveness, coupled with disorder-specific content (4).

A theoretical framework by Watkins and Nolen-Hoeksema (5) proposes that ruminative response to distress first starts as a goal-based coping mechanism aimed to improve an individual’s understanding of his own mental state, but eventually becomes habitual by being repeatedly conditioned with different distressing contexts. This habitual response may then interfere with initiating alternative rewarding strategies which could alleviate one’s negative affect, further perpetuating a detrimental cycle (1, 6, 7). However, the process through which persistent inward-focused rumination may relate to altered initiation of rewarding behavior is not clear.

One potential link between rumination and impaired selection of alternative reward-guided behavior is through altered reward circuitry function. We propose that a habitual tendency for inward-focused processing could influence effective processing of external reward-related stimuli that should normally initiate approach behavior. Approach behavior toward motivationally-relevant stimuli is mediated, at least in part, by the VS/NAcc, a key component in the reward circuitry. A number of animal studies have shown that lesions of the VS/NAcc impair expression of approach responses directed toward, or initiated by, reward-predictive cues (8, 9). Conversely, inducing dopaminergic activity within the VS/NAcc was shown to promote approach behavior (10, 11). Recent human fMRI studies found that different components of motivational approach information (e.g expected reward, expected effort) are also integrated in the VS/NAcc (12, 13). Furthermore, this structure is involved in automatic, habitual responses and initiation of goal-directed instrumental behavior, and is considered to be a “switchboard” between these two approaches (14–16). Variation in reward-related VS/NAcc function as a function of rumination tendencies would provide a first indication for the framework proposed by Watkins and Nolen-Hoeksema (5).

Alternations in VS/NAcc function associated with rumination might also be found during a resting-state fMRI (rsfMRI) scan, traditionally used as a proxy for ruminative-state (17). Other types of repetitive thought patterns, including intrusive-automatic thoughts in obsessive compulsive disorder (OCD) are linked to VS/NAcc-orbitofrontal cortex (OFC) resting-state functional connectivity (rsFC) (18, 19). Previous studies found these automatic thoughts in OCD to be associated with ruminative response style (20, 21), suggesting a more general mechanism might underlie the propensity for rumination.

To test this potential link, we examined associations between trait rumination levels and VS/NAcc function. To this end, we applied a dimensional approach to ruminative tendencies, combining data obtained from a community sample and patients with MDD into a diverse sample (N = 80) exhibiting a broad range of trait rumination scores. We examined two modes of brain function in these participants. First, participants underwent a functional magnetic resonance imaging (fMRI) task involving processing of motivationally-salient monetary gains and losses. Analyses examined associations between rumination tendency levels and VS/NAcc function, and we hypothesized that higher rumination levels would relate to impaired VS/NAcc reward processing. Second, participants completed a rsfMRI scan. Analyses examined associations between rumination tendency levels and rumination-related VS/NAcc intrinsic functional connectivity, with the hypothesis that higher rumination levels would be associated with increased fronto-striatal rsFC.

Analyses controlled for levels of general depressive symptoms in order to isolate effects related specifically to rumination tendencies.

## Methods

### Participants

A total of 80 adult participants (M age = 29.6 years, SD = 9.2; 45 females) took part in the study. This sample included 56 individuals sampled from the community and 24 patients diagnosed with MDD, which enabled a broad range of trait rumination scores to be collected (see below). The non-clinical participants were recruited through social media; inclusion criteria were: age 18-65 years, no self-reported psychiatric or neurological disorders, and no current use of psychotropic/recreational drugs. They were monetarily compensated for their participation. The MDD participants were outpatients of Be’er Ya’akov Mental Health Center and were recruited as part of an interventional study testing add-on repetitive transcranial magnetic stimulation (rTMS) for the treatment of a major depressive episode. A primary diagnosis of MDD was given by trained psychiatrists based on a semi-structured clinical interview using the Structured Clinical Interview for Diagnostic and Statistical Manual of Mental Disorders (22). Data reported here were collected prior to rTMS treatment. These participants were on stable doses of medication during data collection (antidepressants, mood stabilizers, or antipsychotics; see **Table S1** in supplementary material). Patients with a history of drug abuse or dependence within the past year were excluded. All participants completed a gambling task and a rsfMRI scans. We applied a dimensional approach to examine brain activity related to trait rumination, and thus all participants were considered in analyses.

Of the 80 participants, data from nine participants in the rsfMRI and 10 participants in the gambling task were excluded due to significant head movement [> 2.5 mm or >2.5°; rsfMRI: n = 1 (MDD), n = 6 (community sample); gambling task: n = 8 (community sample)], an incidental finding of brain pathology [n = 1 (community sample)], or overweight [n = 1 (MDD)]. The study protocol was approved by the Tel Aviv University, Tel Aviv Sourasky Medical Center and Be’er Ya’akov Mental Health Center institutional review boards and conformed to the Code of Ethics of the World Medical Association (Helsinki Declaration). All participants provided written informed consent prior to participation.

### Psychological Measures

#### Ruminative Response Scale (RRS)

Tendency for ruminative thinking was assessed using the RRS (23), a valid and reliable instrument composed of 22 self-report items, each rated on a scale ranging from 1-4. Items were summed to create a total score reflecting ruminative tendency while dealing with negative affect (total possible scores: 22–88). Cronbach’s alpha in this sample was 0.95. The range of RRS scores in the MDD and community samples was wide (33–82 and 22–70, respectively), as depicted in **Figure S2**.

#### Beck Depression Inventory (BDI)

General depressive symptoms were measured by the BDI (24). The BDI is a 21-item self-report questionnaire. Each item is rated on a scale ranging from 0–3; items were summed to create a total score reflecting severity of depressive symptoms (total possible scores: 0–63). Cronbach’s alpha in this sample was 0.91.

Seven participants did not complete the BDI questionnaire and, of these, two also did not complete the RRS questionnaire properly. These participants were included in analyses performed only on fMRI data but were excluded from analyses relating fMRI and psychological measures.

### Imaging Procedure

**Figure 1:**
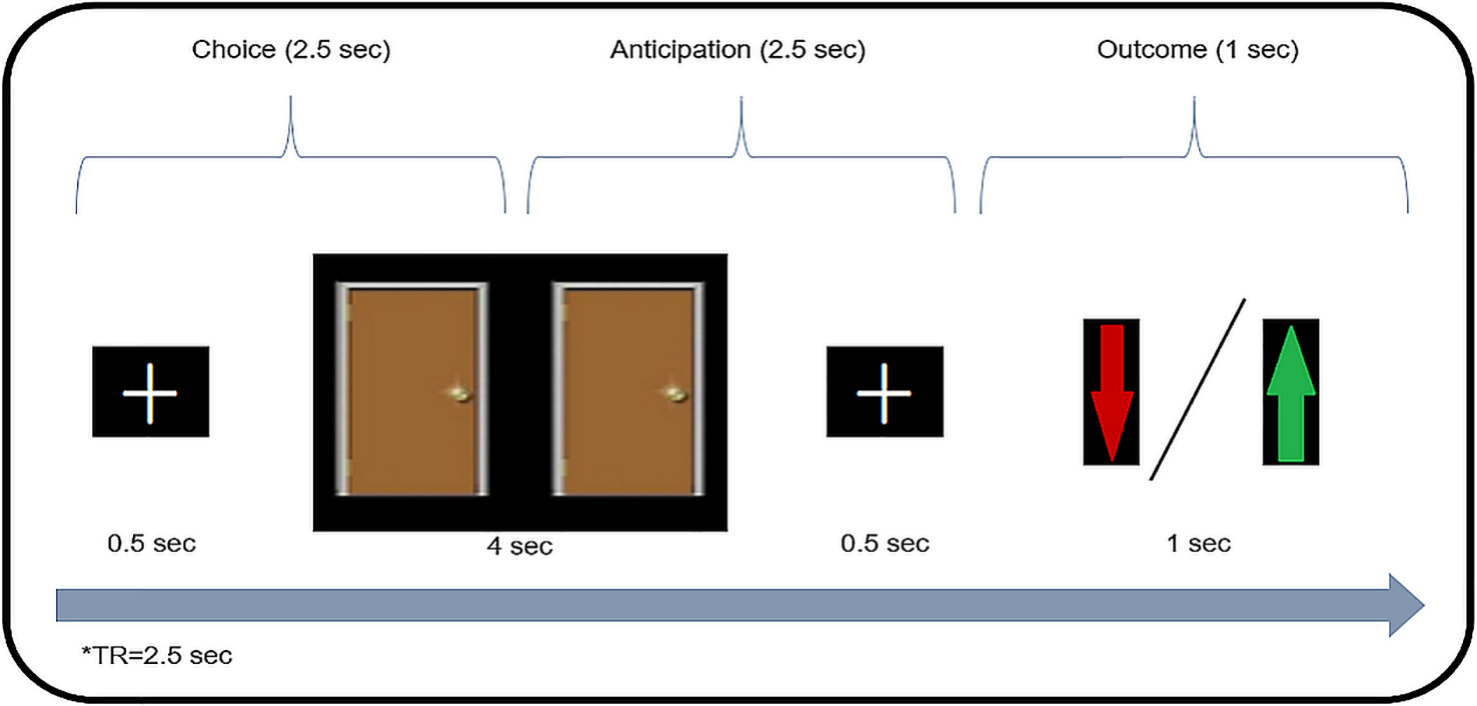
Gambling Task. A schematic illustration of task design. In each trial the participant chooses between two doors (“gambling”), and afterwards is presented with monetary feedback (loss/win).

**Gambling Task.** The gambling task used here was identical to the task reported by Carlson et al. (25) and was performed during scanning. Each trial (see **Figure 1**) started with a white fixation cue presented in the center of a black screen (500 ms). Then, two identical doors were presented side-by-side for 4,000 ms. Participants were instructed before the task that behind one of the doors was a monetary prize [+1 New Israeli Shekel (NIS), equivalent to $0.25] while behind the other door, a loss (−0.5 NIS). Participants used an MRI-compatible response box to choose one of the doors. Participants were instructed that if they did not make their choice while the doors were presented, the computer would select a door at random. Then, after another fixation cue (500 ms), a feedback screen was displayed (1,000 ms) whereby a green arrow indicated a correct guess for a monetary prize, while a red arrow indicated monetary loss. Finally, a blank black screen jittered intertrial interval occurred between each trial (1,500–14,000 ms, M = 4,000 ms).

The task consisted of 60 trials with 30 predetermined wins and 30 losses presented in pseudorandom order and divided equally into two functional runs. That is, unknown to participants, their choice did not influence whether a trial was a win or loss. Prior to the start of the task, participants completed two practice trials featuring one win and one loss. Participants were informed that the sum of their losses and gains would be added to the total experiment payment. The experiment was programmed and run using E-prime software (Psychology Software Tools, Pittsburg, PA).

#### Resting-state fMRI

All participants underwent rsfMRI acquisition. This scan lasted 5 min, and participants were instructed to focus on a cross fixation mark (+) and let their mind freely wander.

### fMRI Data Acquisition

Brain imaging was performed at the Wohl Institute for Advanced Imaging, Tel-Aviv Sourasky Medical Center, using a 3T General Electric Signa Excite scanner (General Electric Medical Systems, Milwaukee Wisconsin) with an eight-channel head coil (N = 35) and a 3T Siemens MAGNETOM Prisma scanner (Siemens, Erlangen, Germany) with a 20-channel head coil (N = 45). 3D anatomical T1-weighted imaging was obtained using SPGR/FLASH sequences with 1 mm iso-voxel. All functional whole-brain scans were performed with gradient echo-planar imaging (EPI) sequence of functional T2*-weighted images. Acquisition parameters for the gambling task were: TR = 2,500ms, TE = 30ms; flip angle = 82°; FOV = 220 × 220 mm; slice thickness = 3mm; no gap; 32/38 interleaved bottom-to-top axial slices per volume for MDD/community sample participants, respectively. Parameters for the rsfMRI were: TR = 3,000ms, TE = 35ms; flip angle = 90°; FOV = 220 × 220 mm; slice thickness = 3mm; no gap; 39/44 interleaved bottom-to-top axial slices per volume for MDD/community sample participants, respectively. Of note, simultaneous electroencephalogram (EEG) recordings were recorded from the community sample participants (data not reported here), leading to fewer slices for safety reasons. Scanner type (GE/Siemens) was controlled for in both task and rsfMRI analyses.

### Data Analysis

Preprocessing procedures were implemented with Statistical Parametric Mapping software (SPM12; http://www.fil.ion.ucl.ac.uk/spm). Functional images for each participant were realigned to the first volume in the time series, slice-timing corrected, co-registered with the analogous anatomical image, normalized into the standard Montreal Neurological Institute (MNI) space, and smoothed with a 6-mm Gaussian kernel. Gambling task data were subjected to SPM12 default high-pass filter cutoff (128 s), and the six-standard motion regressors based on the realignment phase were included as well. Resting-state data were subjected to detrending, bandpass temporal filtering (0.008Hz to 0.15Hz) and CompCor denoising (26), including regression of motion (six regressors and their first derivatives), white matter signal and CSF signal.

Two sets of analyses were conducted to examine the association between trait rumination and VS/NAcc functionality: (a) standard activation during the gambling task. (b) functional connectivity based on rsfMRI data.

Demarcation of Region Of Interest (ROI) in the NAcc was based on peak activation for contrast between reward and punishment obtained from a meta-analysis of reward processing by Liu et al. (27) [MNI] coordinates: (12,8,-4)]. This activation is consistent with the gambling task contrast presented in Carlson et al. (25). Around these coordinates we created a 6 mm sphere using the WFU PickAtlas in SPM12 (28). The right NAcc was chosen since maximal contrast intensity (reward versus punishment) was noted in this structure by both the meta-analysis by Liu et al. (27) and specifically in this task by Carlson et al. (25). Despite the evidence for right laterality, the left VS/NAcc [also based on Liu et al. (27)] was tested as well (MNI coordinates: [-10,10,-4)].

#### Activation

To examine VS/NAcc activation during reward processing, we first defined a first-level model that included four regressors based on the different phases of the task: choice, anticipation, reward and punishment. “Reward” and “punishment” phases were defined according to outcome onset (win and loss, respectively). The period prior to outcome onset was divided into two epochs: The first TR (lasting 2,500 ms) following the doors onset was classified as “choice”, and the following TR was coded as “anticipation”. The “choice” and the “anticipation” conditions were defined as regressors of no interest.

Next, BOLD percent signal change (PSC) within the NAcc was extracted for each participant using the Marsbar ROI toolbox (29). To examine whether rumination tendency levels were associated with reward-related VS/NAcc activation, we calculated the Pearson correlation coefficient between RRS scores and PSC in the VS/NAcc during the reward phase. A similar analysis was conducted on VS/NAcc activation during the punishment phase, to examine specificity of associations to response to reward. Since prior research associates aberrant VS/NAcc activity and depressive symptoms (30), we controlled for BDI scores in analyses.

#### rsFC

To identify brain regions exhibiting functional connectivity (FC) with VS/NAcc that is moderated by ruminative tendency, we performed a whole-brain seed-based FC analysis. This analysis was performed using the CONN toolbox (31), using the NAcc ROI as seed, RRS scores as a covariate-of-interest, and controlling for BDI scores and scanner type. We chose to proceed with the independent meta-analysis-derived right NAcc anatomical seed (and not a functional ROI) in order to minimize the specificity of findings to the sample tested in our particular study.

A number of statistical tests were applied in our analyses. For the gambling task, associations between extracted PSC values and RRS scores were tested with a significance level of 0.05 was used to detect effects. For the whole-brain rsFC analysis, we used a threshold of p < 0.005 (uncorrected) for individual voxels and p < 0.05 (FDR corrected) for cluster extent. All tests were two-sided.

## Results

### Rumination and Depressive Symptoms

The sample exhibited a broad range of RRS scores (22-82; M = 45.1, s.d. = 15) and BDI scores (0–39; M = 13.7, s.d. = 12.6). RRS and BDI scores were highly correlated, *r*(61) = 0.75, *p* < 0.0001.

### Brain Activation

To verify that the chosen VS/NAcc ROI corresponded to activation induced in the task in our sample, we conducted a whole-brain second-level analysis of the reward versus punishment contrast. Importantly, results indicate bilateral VS/NAcc activity, with stronger contrast intensity in the right VS/NAcc [peak MNI coordinates: (12,14,-4)], consistent with previous findings, and overlapping with the a-priori defined right NAcc anatomical mask (see **Figure S1** in the **Supplementary Material**).

**Figure 2:**
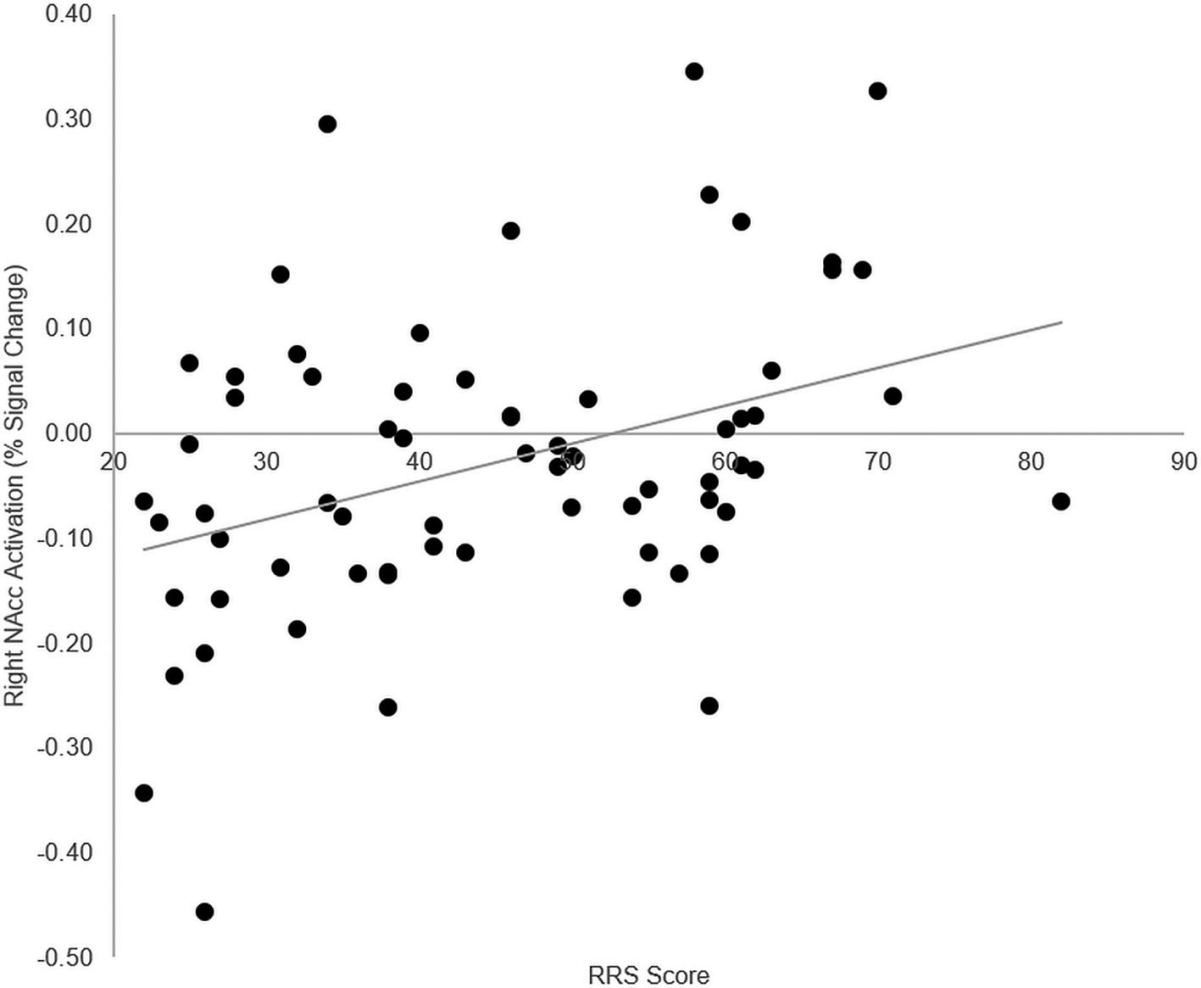
Correlation Between Trait Rumination and VS/NAcc Activation During Reward. VS/NAcc activity as a function of RRS scores (n = 68). Positive correlation between VS/NAcc activation during reward processing and RRS scores. This correlation remains significant while controlling for BDI scores and scanner type [*r*(60) = 0.27, *p* = 0.03]. VS, ventral striatum; NAcc, nucleus accumbens; RRS, Ruminative Response Scale; BDI, Beck Depression Inventory.

To examine relations between VS/NAcc activity and trait rumination, we extracted mean BOLD PSC from the NAcc ROI, separately for the reward and punishment conditions, and correlated these with RRS scores. RRS scores correlated positively with reward-related [right VS/NAcc: *r*(66) = 0.37, *p* = 0.002; left VS/NAcc: *r*(66) = 0.28, *p* = 0.02] and punishment-related [right VS/NAcc: *r*(66) = 0.27, *p* = 0.03; left VS/NAcc: *r*(66) = 0.25, *p* = 0.04] processing in the VS/NAcc. After controlling for overall depressive symptoms (BDI scores) and scanner type, only the correlation with reward activation within the right VS/NAcc remained significant, *r*(60) = 0.27, *p* = 0.03 (right VS/NAcc, punishment: *r*(60) = 0.18, *p* = 0.16; left VS/NAcc, reward: *r*(60) = 0.13, *p* = 0.31, punishment: *r*(60) = 0.08, *p* = 0.54). This association is depicted in **Figure 2**.

### rsFC

**Figure 3:**
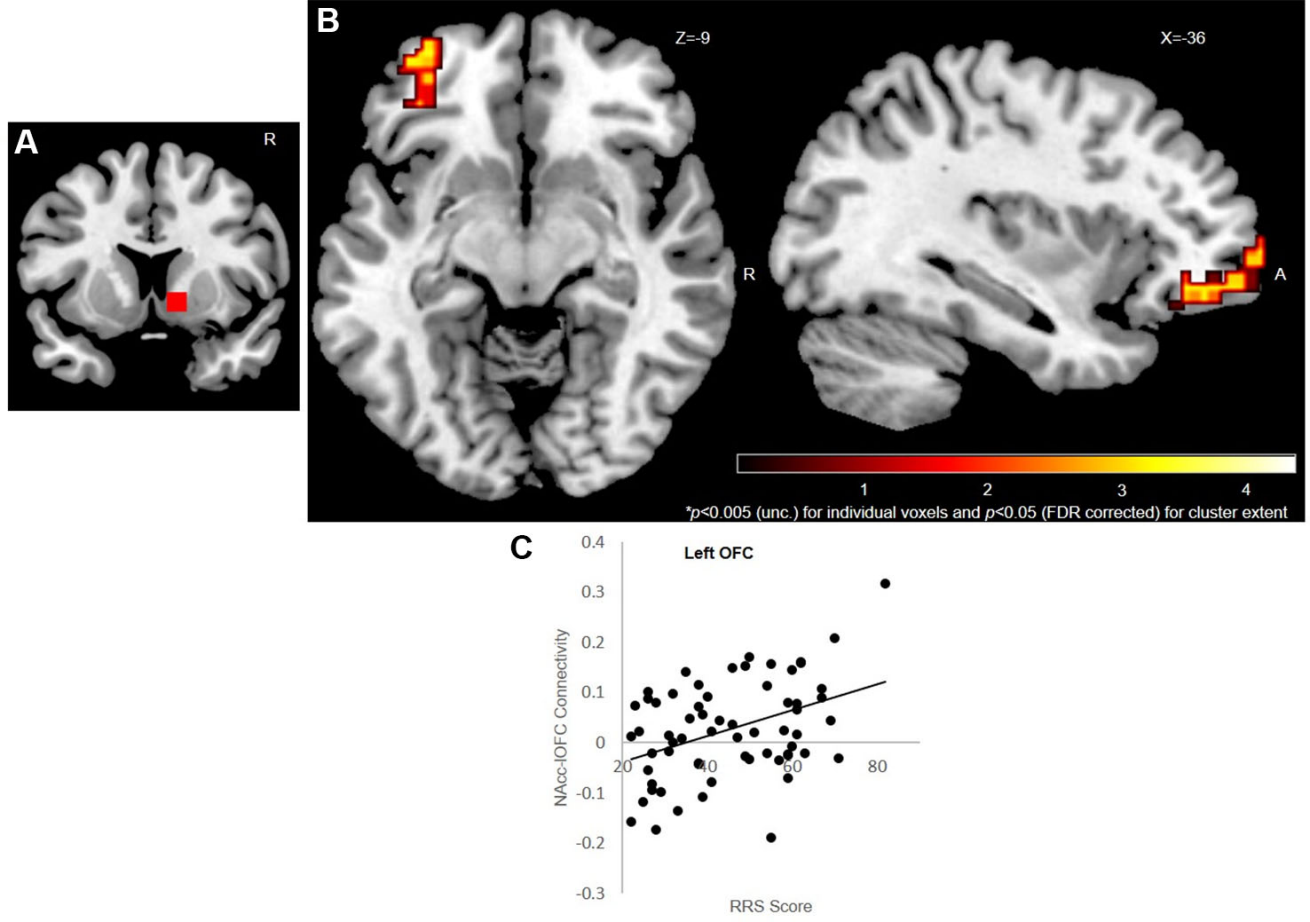
VS/NAcc-OFC rsFC. Seed-based [right NAcc **(A)**] resting state functional connectivity results (n = 64). Left OFC **(B)** connectivity with the right NAcc is positively correlated with ruminative tendency (while controlling for BDI levels and scanner type). Results displayed at *p* < 0.005 (uncorrected) for individual voxel and *p* < 0.05 (FDR corrected) for cluster extent. Color bar reflects t scores. **(C)** Illustration of Z connectivity values as a function of RRS scores. VS, ventral striatum; NAcc, nucleus accumbens; rsFC, resting state functional connectivity; OFC, orbitofrontal cortex; BDI, Beck Depression Inventory; RRS, Ruminative Response Scale.

Whole-brain, seed-based FC analysis for the right NAcc seed revealed left OFC rsFC was associated with trait rumination scores [see **Figure 3** and **Table S2** in the **Supplementary Material**; peak MNI coordinates: (-24,62,-2)]. Importantly, this fronto-striatal enhanced connectivity has previously been implicated in OCD patients. The left NAcc seed did not reveal any findings.

Additionally, we conducted post-hoc analyses in which we tested the associations between RRS scores and VS/NAcc activation and connectivity separately within the community sample and the MDD sample. Briefly, the functional connectivity results were similar across groups whereas the activation results were significant only in the healthy sample; see **Supplementary Material** and **Figure S3** or **Figure S4** for more information.

## Discussion

### Main Findings

The goal of this study was to examine the relation between trait rumination and VS/NAcc functionality. Our results revealed a positive correlation between trait rumination and VS/NAcc response to reward and increased ruminative-dependent rsFC of the VS/NAcc with the left OFC. These results link ruminative tendency with reward-related and rsfMRI VS/NAcc activity and connectivity, respectively, suggesting a role for this subcortical structure and reward-related processing in ruminative thinking.

Of note, associations between rumination severity and resting-state functional connectivity were evident across the sample as well as separately within the MDD and community groups. However, associations between rumination severity and VS/NAcc activation during the task emerged across the sample and in the community group, but not in the MDD group. Although the aim of this study was not to compare MDD patients and healthy controls, but rather examine correlates of rumination severity across a wide continuum, the absence of an activation effect in the MDD group deserves consideration. The absence of a significant correlation in the MDD group may be due to the smaller sample size in the MDD group (n = 23) as well as the restricted range of the RRS scores when considering this group alone, as most of the MDD patients were characterized by high rumination scores, thereby limiting statistical power to detect associations separately within groups. Furthermore, the presence of a medical condition could have also affected the presence of this association. Additional studies on larger samples of MDD patients could examine the replication of these findings and further explore this differential pattern of associations. Alternatively, acquiring data from individuals with high rumination levels but who are not diagnosed with MDD could help resolve this differential effect and the potential confound of MDD diagnosis.

### Comparison With Findings From Prior Studies

To date, VS/NAcc functionality has not been considered a key element in the neural circuitry subserving rumination. However, the interference between ruminative thinking and initiation of rewarding strategies suggests this thinking pattern might be reflected in the functionality of this region. The gambling task results revealed a positive correlation between VS/NAcc reward processing responsivity and rumination. Such an association was contrary to our prior expectation that diminished VS/NAcc activation is associated with ruminative tendencies. A number of explanations might account for this finding. First, striatal dopaminergic activity in response to reward has been shown to reflect not only the absolute value of an outcome, but whether it is better or worse than expected, i.e., a prediction error (32, 33). Given our hypothesis about an internal focus of attention in high-ruminators, it is possible that a tendency for greater rumination interferes with calculation of expected values, leading to greater reward prediction errors. Moreover, the OFC has been linked to reward valuation processes (34, 35), and evidence for perturbed rumination-OFC function has been identified in our study. More specifically, chronic maladaptive low expectation could be a potential mechanism for the lack of approach behavior associated with ruminative tendency. Another potential explanation for this finding considers the distinction between phasic and tonic dopaminergic activity. While phasic dopaminergic release is induced by task events, tonic dopaminergic release is expected to be relatively constant (36). Both physiological mechanisms are known to affect motivation and learning processes (37), and VS/NAcc fMRI BOLD signal is known to be associated with dopamine levels (38). Differential BOLD response to reward could reflect differences in phasic (events) to tonic (baseline) ratios of dopamine activity. These two potential explanations could be tested in future studies.

Our rsfMRI findings complement the task results, indicating that ruminative tendency is associated with increased fronto-striatal rsFC which is not task-dependent. This increased VS/NAcc-OFC connectivity is a consistent neuronal marker in OCD (18, 19). Considering our results together with this well-known neuroimaging finding in OCD, suggest that this increased connectivity may underlie repetitive-intrusive thinking and/or promote general propensity for these thinking patterns. This understanding supports the conceptualization of rumination as a part of repetitive negative thinking phenomenon, implying that a general cognitive mechanism might underlie this thinking pattern.

Our results extend prior research, studying effects related to rumination outside the traditional framework of rumination induction. Such an approach may shed light on the potential influence of rumination tendencies on neural functions that engage overlapping, or associated, circuitry. Here, such an association manifested in VS/NAcc function which exhibited relations with rumination levels both in terms of response to reward and rsFC, which may in turn impede adaptive response selection. Additional research is needed to further explore these associations.

Our findings also correspond with dual-system theories of behavioral control. According to this theory, behavioral control is parsed between a goal-directed “model-based” and more habitual “model-free” system, while the balance between these two is posited to depend on VS/NAcc activity (39, 40). A recent large-scale study applied a computational approach to characterizing different psychiatric symptom dimensions related to deficits in goal-directed control. This study found that symptom dimensions comprising compulsive behavior and intrusive thought to be the most strongly associated with deficits in goal directed control (41). Our findings suggest that VS/NAcc functionality may relate to this association. Finally, the VS/NAcc functional switchboard perspective (14–16) adopted in our study can also be integrated with the findings of Whitmer and Gotlib (42) regarding rumination and attention switching. In their paper, the cognitive switching process was divided into two components: an inhibitory switching process (i.e., deactivation of the no longer relevant task), and noninhibitory switching process (i.e., activation of mental representation of the new task). While trait rumination was found to be associated with impaired inhibitory switching process, state rumination was found to be associated with noninhibitory switching process. Inspired by these notions, we can think about state rumination as a “task” that should be inhibited and might be correlated with the fronto-striatal circuits revealed in our rsfMRI, and the noninhibitory switching as a process guided by reward processing which might be correlated with our findings in the gambling task.

### Limitations

Several limitations in the present study should be considered. First, the majority of patients with MDD were receiving pharmacological treatment. Future studies may aim to include medication-free participants. Second, the design of the gambling task did not allow us to reliably examine outcome anticipation processes relevant to habitual tendencies (43). We sought to follow the exact experimental design by Carlson et al. (25) in which participant’s choice between doors was not limited to a specific timeframe, therefore choice and anticipation phases could not be distinguished. Finally, a post-hoc analysis of task activation and rumination severity revealed insignificant results for the MDD group (see **Figure S3** in supplementary material). The absence of this association may reflect the smaller sample size in the MDD group (n = 23) as well as the restricted range of the RRS scores when considering this group alone, and calls for additional research using larger groups or individuals with high rumination levels but no MDD diagnosis.

### Conclusions and Future Directions

To our knowledge, this is the first study to examine the interference between the persistent inward focus of rumination and initiation of rewarding strategies using neuroscientific tools. This led us to focus on the VS/NAcc, a region not typically studied with regard to rumination. Our findings revealed a positive correlation between trait rumination and VS/NAcc response to reward and increased fronto-striatal ruminative-dependent rsFC. Further studies will enable a better understanding of the directionality between these processes. Moreover, inclusion of additional clinical populations with elevated rumination scores (e.g., OCD) will provide a better understanding about the transdiagnostic nature of these findings. Future studies should also strive to recruit larger samples, particularly of MDD patients, to increase statistical power. Lastly, effective behavioral measurements in future studies could help determine the way these neuronal findings correspond with habitual tendencies and formation of alternative rewarding strategies as well as further illuminate depressive-rumination using this theoretical perspective.

## Data Availability Statement

The datasets collected for this study will not be made publicly available. We did not obtain consent from our participants for sharing of data when the study was conceived, so we cannot provide raw participant-level data.

## Ethics Statement

The studies involving human participants were reviewed and approved by Tel Aviv University, Tel Aviv Sourasky Medical Center and Be’er Ya’akov Mental Health Center institutional review boards. The patients/participants provided their written informed consent to participate in this study.

## Author Contributions

AE and RA contributed equally to writing the manuscript, data acquisition, and data analysis. IJ, MA, and TG contributed to data acquisition, a significant amount of data analysis, and consulted on study design. KA, EA, and HL conducted clinical assessments and clinical screenings of the subjects. EG-S contributed to the writing of the manuscript and management of the study team. TH and EH designed the study and oversaw all aspects of data acquisition, data analysis and the writing of the manuscript.

## Funding

This work is part of the BSMT consortium (Brain Stimulation and Monitoring Toolbox), and was supported by grants from the MAGNET program of the Israeli Innovation Authority (IIA).

## Conflict of Interest

The authors declare that the research was conducted in the absence of any commercial or financial relationships that could be construed as a potential conflict of interest.
