# Supplementary material for: Ruminative Tendency Relates to Ventral Striatum Functionality: Evidence From Task and Resting-State fMRI
Source: Front Psychiatry. 2020 Feb 20;11:67. doi: 10.3389/fpsyt.2020.00067 (PMC7044182; doi:10.3389/fpsyt.2020.00067)
Supplement: Supplementary file 1 [file DataSheet_1.pdf]

Supplementary Material

Additional Information About MDD Patients

Patient Number	Gender	Age	CGI-Severity	HDRS-21	List of medications	Currently on medication	Number of past hospitalizations
1	F	29	6=severely ill	24	Fluoxetine 40mg	Yes	0
2	F	58	5=markedly ill	29	Bupropion 150mg, Fluoxetine 40mg	Yes	0
3	M	42	5=markedly ill	27	Nortriptyline 150mg, Bupropion 150mg	Yes	2
4	M	41	4=moderately ill	25	Mianserin 60mg	Yes	0
5	M	33	4=moderately ill	25	Escitalopram 20mg, Lorazepam 1mg	Yes	0
6	M	60	4=moderately ill	25	Amitriptyline 150mg, Lorazepam 2mg, Mirtazapine 30mg	Yes	0
7	F	49	6=severely ill	25	Aripiprazole 10mg, Clonazepam 1mg	Yes	0
8	M	47	5=markedly ill	25	Escitalopram 40mg, Clonazepam 1.5mg, Olanzapine 20mg, Amitriptyline 200mg	Yes	0
9	F	56	5=markedly ill	25	Milnacipran 50mg	Yes	0
10	M	24	4=moderately ill	25	Licarbium 1350mg	Yes	0
11	F	39	5=markedly ill	25	Quetiapine 100mg, Clonazepam 0.5mg, Fluoxetine 50 mg, Topiramate 25mg	Yes	1
12	M	35	3=mildly ill	25	Escitalopram 20mg	Yes	0
13	M	34	4=moderately ill	26	Venlafaxine 150mg, Perphenazine 4mg	Yes	0
14	M	42	4=moderately ill	25	Vortioxetine 20mg, Aripiprazole 5mg	Yes	1
15	M	31	6=severely ill	25	Clomipramine 300mg, Escitalopram 20mg, Duloxetine 30mg	No	0
16	M	29	4=moderately ill	25	Sertraline 100mg, Vortioxetine 10mg	Yes	0
17	M	27	4=moderately ill	25	Vortioxetine 20mg, lamotrigine 400mg	Yes	1
18	M	54	5=markedly ill	27	Fluvoxamine 150mg, Aripiprazole 5 mg	Yes	0
19	F	28	4=moderately ill	not reported	Venlafaxine 150mg, Clonazepam 1mg	No	0
20	M	43	4=moderately ill	not reported	Vortioxetine 20mg, Quetiapine 100mg	Yes	0
21	F	30	4=moderately ill	31	Venlafaxine 150mg	Yes	0
22	F	53	4=moderately ill	32	Venlafaxine 150mg, Clonazepam 1mg	Yes	0
23	M	38	4=moderately ill	25	None	No	0

Table S1. Demographic, clinical and drug information about MDD patients. The clinical assessments included the clinical global impression (CGI) scale (Bunser and Targum, 2007; Guy, 1976), and Hamilton Depression Rating Scale (HDRS; Hamilton, 1960).
Note: MDD=major depressive disorder

Reward>Punishment

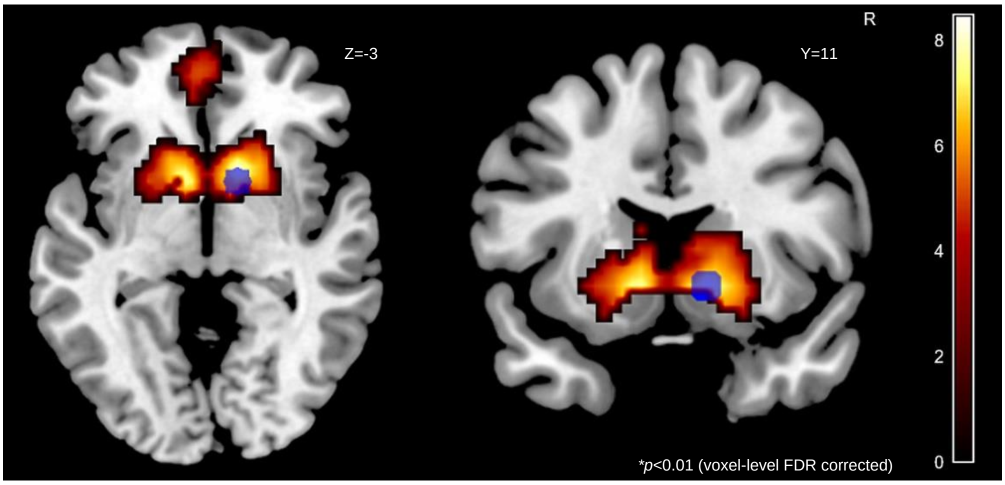

Figure S1. Whole brain analysis fMRI results of the gambling task (n=70) overlap with right NAcc anatomical mask (blue). Reward>Punishment contrast revealed bilateral VS activity. Results displayed at a significance threshold of p<0.01 (voxel-level FDR corrected) and a minimum cluster size=50 for visualization purposes. Color bar reflects t scores. Only positive effects survived whole brain corrections.
Note: NAcc=nucleus accumbens; VS=ventral striatum.

VS/NAcc-OFC rsFC

Brain Region	MNI Coordinates			Size *p*-Unc.	Peak *p*.-Unc.	Voxels
Increased connectivity	X	Y	Z			
Left OFC	-24	62	-2	<0.001	<0.001	497

Table S2. Left OFC showing increased functional connectivity with NAcc during RS as a factor of rumination levels. Results are based on a threshold p<0.005 (unc.) for individual voxel and p<0.05 (corrected) for cluster extent.
Note: VS=ventral striatum; NAcc=nucleus accumbens; rsFC=resting-state functional connectivity; RS=resting-state; OFC=orbitofrontal cortex.

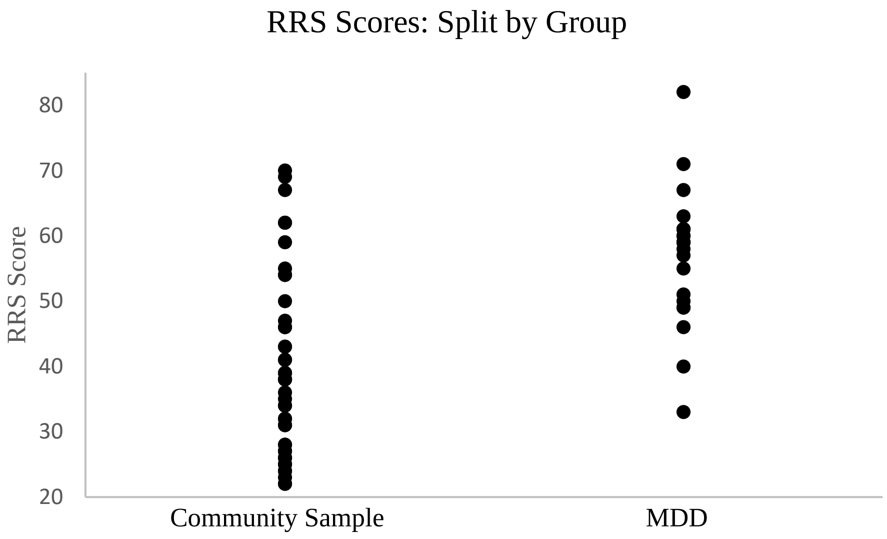

Figure S2. RRS score distribution in the community sample and the MDD groups.

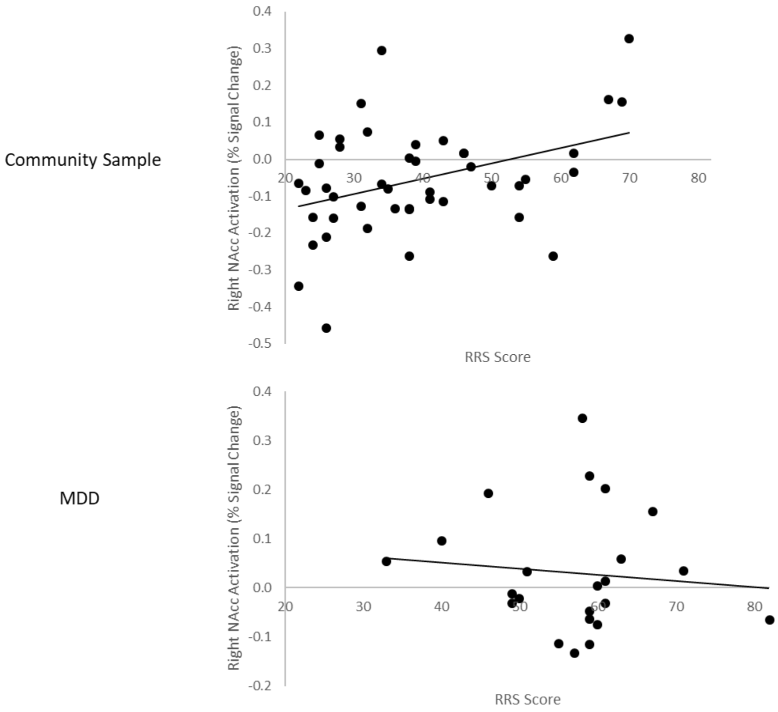

Figure S3. VS/NAcc activity as a function of RRS scores: split by group. Positive correlation between VS/NAcc activation during reward processing and RRS scores was found in the community sample group (n=45). This correlation remains significant while controlling for BDI scores and scanner type ($r(36)=0.41$, $p=0.01$). Correlation when only the MDD group was considered didn't yield significant results.

Note: VS=ventral striatum; NAcc=nucleus accumbens; RRS=Ruminative Response Scale; BDI=Beck Depression Inventory; MDD=Major Depressive Disorder.

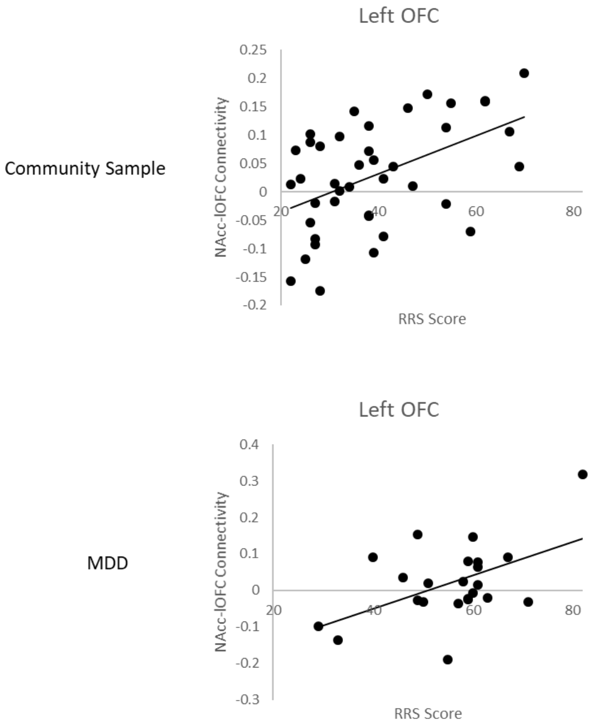

Figure S4. Resting-state Z connectivity values as a function of RRS scores: split by group. Left OFC connectivity with the right NAcc is positively correlated with ruminative tendency in both groups (while controlling for BDI levels and scanner type). Community sample: *r*(39)=0.49, *p*=0.001; MDD: *r*(21)= 0.51, *p*=0.013.
Note: NAcc=nucleus accumbens; OFC=orbitofrontal cortex; BDI=Beck Depression Inventory; RRS=Ruminative Response Scale.

<u>Figures S3 and S4-note:</u>

In exploratory post-hoc analyses, we also examined the associations between RRS scores and VS/NAcc activation and connectivity separately within the community sample and the MDD sample (see Figures S3 and S4 above). For activation data, similarly to the analysis on the full sample, a significant correlation emerged in the community sample group, *r*(36)=0.41, *p*=0.01; however, no significant correlation was observed in the MDD sample, *r*(20)=-0.01, *p*=0.96. For connectivity data, a significant correlation emerged both in the community sample group and the MDD group (community sample: *r*(39)=0.49, *p*=0.001; MDD: *r*(21)=0.51, *p*=0.013).

References

Busner J and Targum SD., (2007). The clinical global impressions scale: applying a research tool in clinical practice. Psychiatry (Edgmont) 4, 28–37.

Guy W., (1976). ECDEU Assessment Manual for Psychopharmacology. Rockville, MD: US Department of Health, Education, and Welfare Public Health Service Alcohol, Drug Abuse, and Mental Health Administration.

Hamilton M., (1960). A rating scale for depression. Journal of Neurology, Neurosurgery and Psychiatry 23, 56–61.
